# A simple flash sintering setup under applied mechanical stress and controlled atmosphere

**DOI:** 10.1016/j.mex.2015.10.004

**Published:** 2015-10-17

**Authors:** L.B. Caliman, E. Bichaud, P. Soudant, D. Gouvea, M.C. Steil

**Affiliations:** a Department of Metallurgical and Materials Engineering, Escola Politécnica, Universidade de São Paulo, Av. Prof. Mello Moraes n. 2463, 05508-030 São Paulo, SP, Brazil; b Laboratoire d’Electrochimie et de Physicochimie des Matériaux et des Interfaces (LEPMI) – UMR 5631 CNRS-Université de Grenoble Alpes, BP 75, 38402 St Martin d’Hères, France

**Keywords:** A simple flash sintering setup under applied mechanical stress and controlled atmosphere, Current assisted sintering, Flash sintering setup, Atmosphere control, Mechanical stress application

## Abstract

Most flash sintering experimental set-ups use dog bone-shaped specimen and DC current, which results in heterogeneously distributed densification and grain growth throughout the sample. This is the reason why only the sample's core characteristics after flash are taken into account. On the other hand, some recent procedures suggest the use of cylindrical pellets, which have some advantages compared to the traditional mode as the use of easily conformed samples and its final uniformity.

Our new experimental set-up offers the possibility of atmosphere control and pressure application. Also the electrodes material change can be easily made when necessary. Shrinkage measurements and impedance spectroscopy are realized in situ and experimental parameters, as oven heating for example, can be varied to control microstructure changes.

Advantages:

• Sample can be entirely recovered at the end of the experiment and can be analyzed throughout its entire extension, including regions in contact with the electrodes that may present some differences from pellets inner part.
• The use of AC current enables the study of different frequencies effects.
• Experimental set-up can be adapted to different kinds of electrolytes (samples), easily changing electrode's material and atmosphere.

## Graphical abstract

**Figure:**
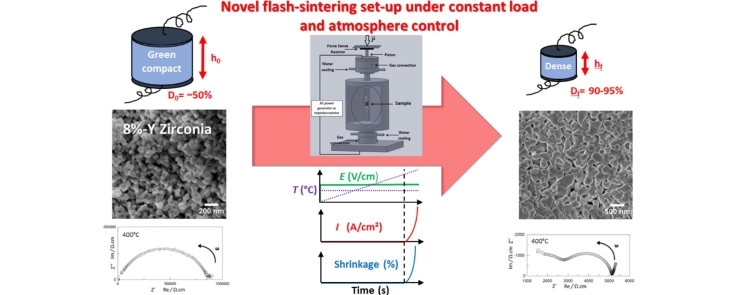

## Methods details

Flash sintering is a current assisted sintering technique able to densify samples in short periods of time (as fast as 5 s) and most importantly, at furnace temperatures significantly lower than in conventional sintering [1], [2]. It was firstly reported for yttrium-stabilized zirconia and later it has been proved successful for a large range of ceramic oxide materials [3], [4], [5], [6], [7], [8].

Traditional flash sintering experiments use a dog-bone shaped sample hanged in the furnace center by two metallic (platinum) wires which act simultaneously as electrodes and sample's support. Some new procedures, including one of ours [2], [9], use easily pre-conformed pellet-shaped samples placed between two platinum discs electrodes. The sample's only contacts are the electrodes, so is mandatorily for the current to pass through the ceramic, which is the flash sintering technique principle.

The flash sintering is characterized by an abrupt increase in the current density through the sample, accompanied of shrinkage and densification. Samples are required to be slightly conductive in order to allow current flow and since each type of electrolyte has a different conduction mechanism, platinum electrode and oxygen-rich atmosphere may not be suitable for all kinds of electrolytes.

We developed a new experimental set-up that allows atmosphere change and control, the appliance of a mechanical pressure to improve densification, and also the electrode material change when necessary.

Despite any SPS (spark plasma sintering) similarities, our set-up do not involve the use of dies, forcing current to pass through the sample; uses much smaller pressures; uses pre-conformed samples; allows the use of the desired atmosphere; and the most important characteristic: flash sintering allows densification to happen in just a few seconds.

### Sample preparation

Samples are uniaxial and isostatic cold pressed under 60 and 250 MPa, respectively, resulting in green relative densities of about 50–55% TD. Pellets form and dimensions are optional as long as there are flat surfaces with enough area to insure a good contact with the electrodes.

Set-up electrodes discs and wires are made of platinum. Sample is recovered with a layer of a metallic ink in both flat surfaces to insure intimate contact. Platinum grids can also be used to improve contact between set-up electrodes and electrode layer in the sample.

An interesting aspect of using a pellet sample instead of a dog-bone shaped sample is that the electrode material can be changed (changing the metallic ink layer between the ceramic and the platinum electrode) to ensure the electrical and electrochemistry compatibility between the electrode and sample material.

### Set-up description

**Fig. 1:**
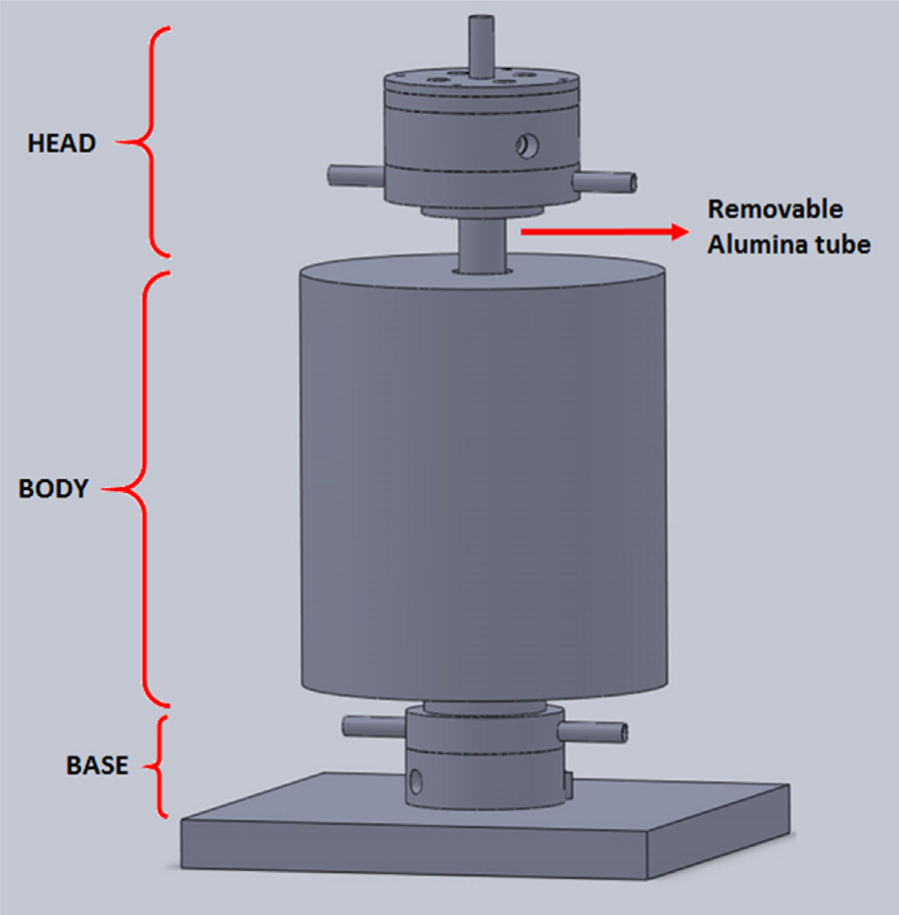
External view of flash sintering furnace set-up.

Flash sintering set-up consists in a vertical furnace where the sample is located in the center of an alumina tube, only in contact with both electrodes (grids optional). The set-up is divided in three main detachable parts: head, body (consisting of cylindrical furnace and removable alumina tube) and base, Fig. 1.

**Fig. 2:**
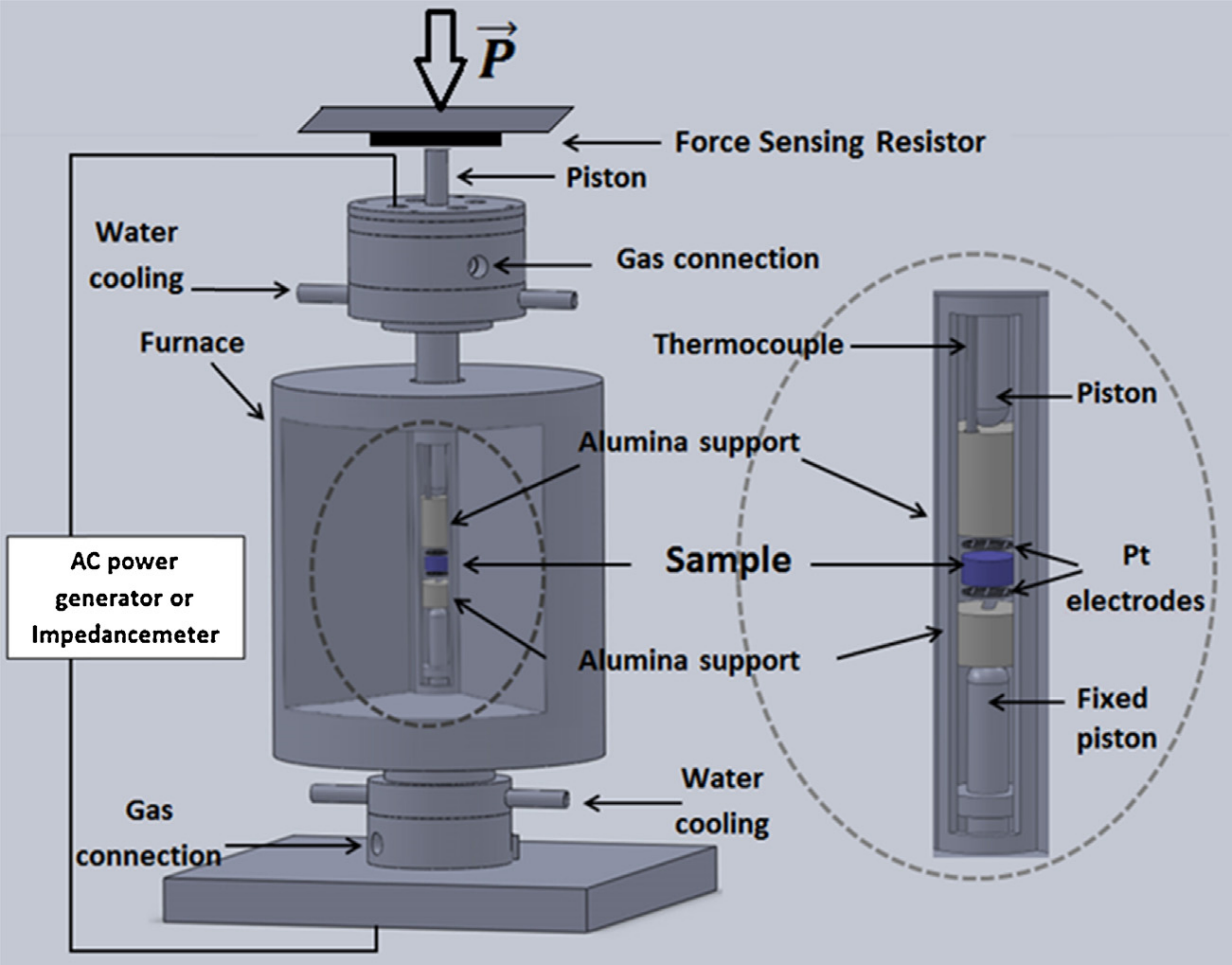
Flash sintering experimental set-up and details.

Base has an independent water cooling system, a gas connection and electrical contacts. A fixed piston supports the bottom electrode, its alumina base and the sample, as it can be seen in Fig. 2.

Body is composed by the cylindrical vertical furnace and a removable alumina tube. When the alumina tube is removed, it reveals the assembly fixed on the base.

**Fig. 3:**
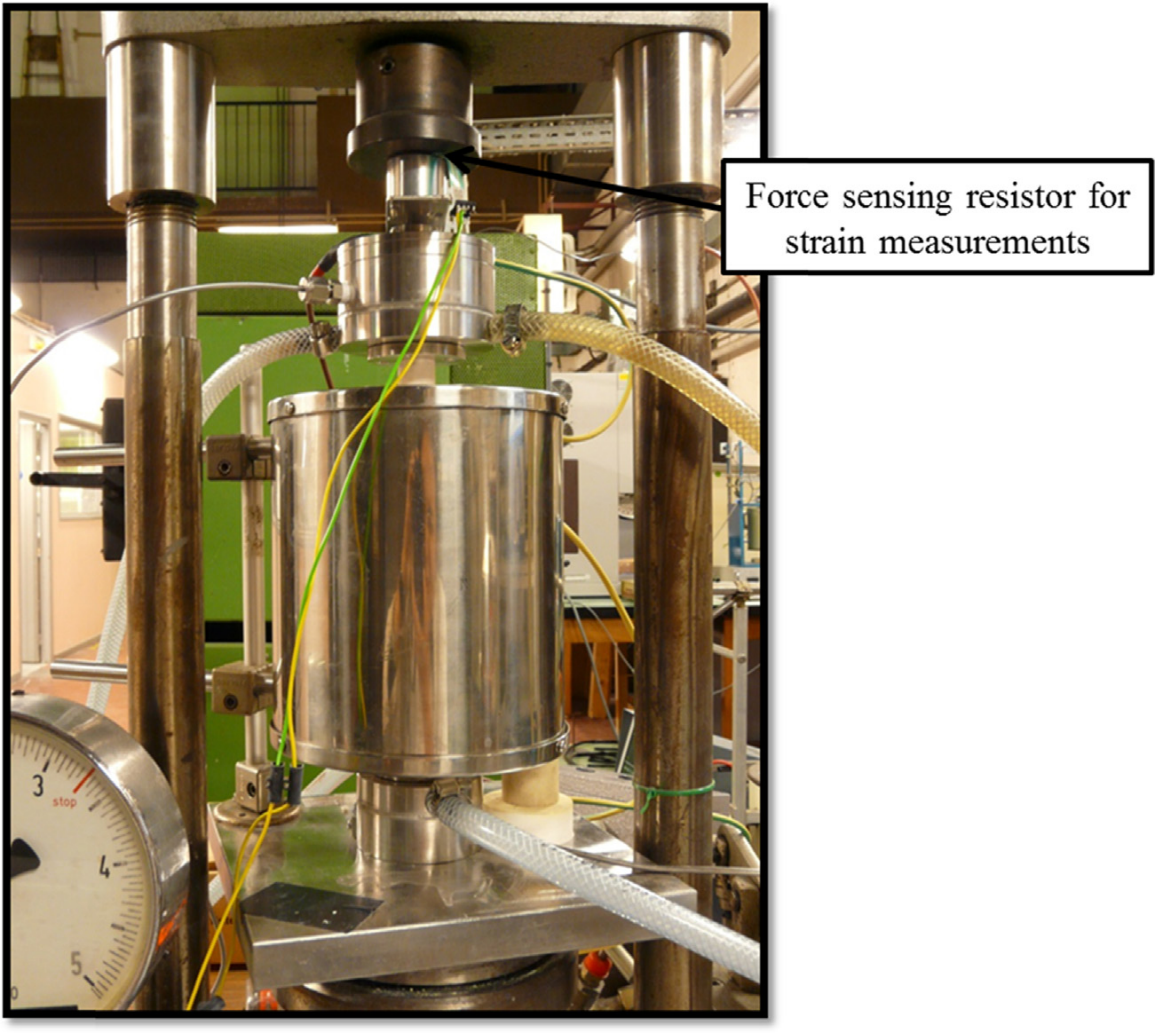
Functioning set-up placed in the uniaxial press.

Head is the last part of the set-up put together. It contains the second platinum electrode, a thermocouple and a free-moving piston that touches the electrode's alumina support in one extremity. Head also has independent water cooling system, a gas connection and electrical contacts. Located in the external extremity of the piston there is a pressure sensor (SFR – force sensing resistor type sensor) that allows controlled mechanical stress application. The experimental set-up is placed in a uniaxial press for load application as show in Fig. 3. The force sensing resistor is placed between the free-moving piston of the head and the press piston.

### Method description

The AC voltage was provided by a Pacific Smart Source 115ASX AC power generator connected to the sample holder and controlled by the UPC Manager V.1.4 software. Key parameters that control the process can be independently determined:

• Signal nature and frequency (DC or AC): from 0 to 1000 Hz.
• Voltage (electrical field applied to the sample): from 1 V to 120 V.
• Maximum current achieved (current density trough the sample): up to 15 A.
• Switch from voltage control to current control once settled maximum current is achieved.
• The data log system continuously registers voltage, current, and power evolution as function of time.

Also, power source is capable of a very quickly (0, 1s) switch from voltage control to current control once the pre-settle current is achieved. In current control mode, equipment can be programed to either discontinue all power when maximum current is achieved, or maintain a certain pre-settled current during a determined period of time.

The set-up offers many other possibilities as: the use of different oxygen pressures during flash sintering experiments; the maintenance of current density for a period after flash; and the interruption of the electrical field after flash to compare initial and final microstructures.

**Fig. 4:**
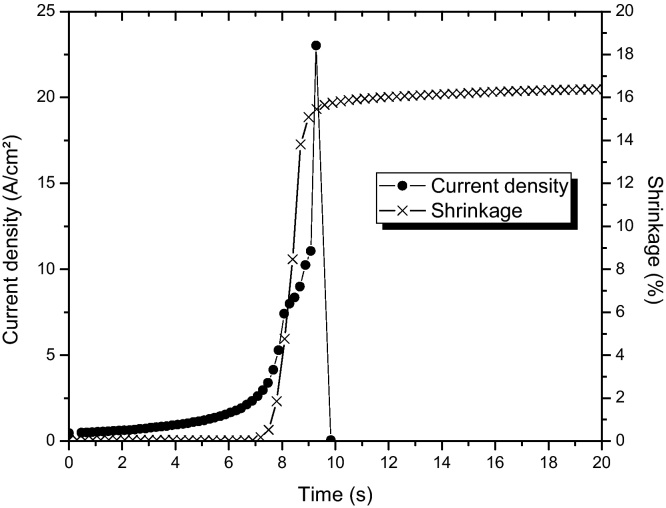
Current density and shrinkage evolution during 8%Y-zirconia isothermal flash sintering (900 °C and 100 V/cm).

In isothermal flash sintering, an electrical field is applied to a sample while furnace temperature is kept constant [2], [10], [11], [12]. Flash onset is observed after a delay time, which is dependent of the sample's initial conductivity. The delay time is influenced by the term $E_{0}^{2}\sigma_{0}$, i.e., the resultant Joule power density supplied, as shown previously by Bichaud et al. [10]. Fig. 4 shows one example of YSZ ceramic isothermal flash sintering experiment at 900 °C.

## Some method results

### Different atmospheres and electrode material

Beta-alumina flash sintering has shown that different ionic conductors require specific conditions to happen.

Platinum is commonly used as electrode material for its compatibility with oxygen conductors such as zirconia. Electrode reaction is shown in the following equation.

(1) $$\frac{1}{2}{\text{O}}_{2}+2{\text{e}}^{-}↔{\text{O}}^{2-}$$

**Fig. 5:**
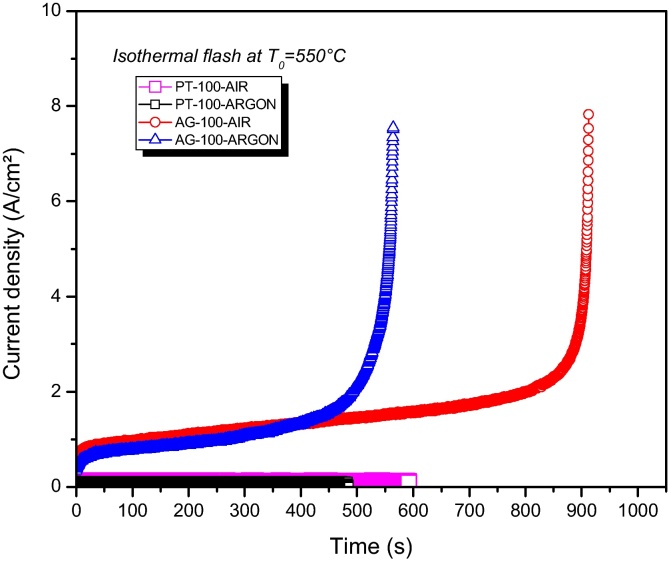
Beta-alumina flash sintering attempts (at 550 °C) with different electrode materials and atmospheres.

When the charge carriers are other species like Li^+^ or Na^+^ (as in beta-alumina), platinum does not allow current flow since there is no charge exchange between the electrode and electrolyte (ceramic). That kind of behavior can be seen in Fig. 5 for samples PT-100-AIR (Pt electrode, 100 V/cm electrical field in air) and PT-100-ARGON (Pt electrode, 100 V/cm electrical field in argon atmosphere).

Beta-alumina is a sodium conductor and it flash sintering was only possible when silver was used as the electrode material. For AG-100-AIR (Ag electrode, 100 V/cm electrical field in air), the long delay time and oxygen-rich atmosphere caused the material partial decomposition. Better results were obtained with sample AG-100-ARGON (Ag electrode, 100 V/cm electrical field in argon atmosphere) using argon as the atmosphere (Fig. 5). Final sample presented retraction, densification, low resistance and non-altered chemical composition.

### Pressure appliance

Pressure-assisted sintering shows some advantages compared to conventional.

**Fig. 6:**
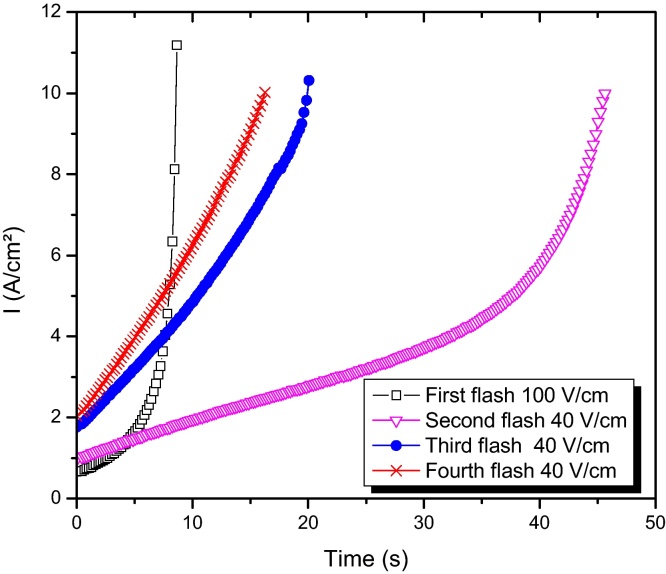
Multiple flash sintering of 3%Y-zirconia: an initial flash (without load application) consolidates the sample enough to endure the pressure appliance in the subsequent flashes, each one with a 48 MPa load (40 V/cm at 800 °C).

**Fig. 7:**
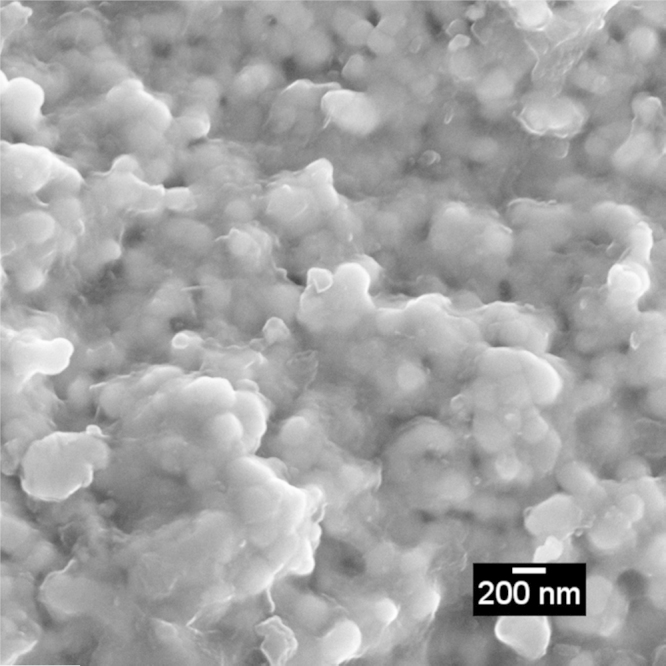
SEM micrograph of multiple flash sintered 3%Y-zirconia ceramics.

A multiple-flash was performed in a 3%Y-zirconia sample to demonstrate pressure influence in the flash sintering, Fig. 6. A first rapid pressure-free flash was realized to improve sample's mechanical resistance in order to endure further pressure appliance (electrical field 100 V/cm at 800 °C). Subsequent flashes with a 48 MPa charge each (40 V/cm at 800 °C) improved densification as shown clearly by the final material micrograph (Fig. 7).
